# Integrated Plan of Insecticide Resistance Surveillance in Mosquito Vectors in France

**DOI:** 10.3390/insects14050457

**Published:** 2023-05-12

**Authors:** James Devillers, Jean-Philippe David, Benoit Barrès, Haoues Alout, Bruno Lapied, Sébastien Chouin, Isabelle Dusfour, Catherine Billault, Farida Mekki, Isabelle Attig, Vincent Corbel

**Affiliations:** 1 CTIS, 69140 Rillieux-La-Pape, France; 2 Laboratoire d’Ecologie Alpine, UMR UGA-USMB-CNRS 5553, Université Grenoble Alpes, 38041 Grenoble Cedex 9, France; 3 Université de Lyon, Anses, INRAE, USC CASPER, 69364 Lyon Cedex 7, France; 4 ASTRE, UMR117 INRAE-CIRAD, 34398 Montpellier Cedex 5, France; 5 Université Angers, INRAE, SIFCIR, SFR QUASAV, 49045 Angers Cedex, France; 6 Conseil Départemental de la Charente-Maritime, DEM, Démoustication, 17076 La Rochelle, France; 7 Institut de Recherche pour le Développement (IRD), MIVEGEC, Univ. Montpellier, CNRS, IRD, 34394 Montpellier, France; 8 Anses, DEPR, 94701 Maisons-Alfort Cedex, France; 9 Laboratório de Fisiologia e Controle de Artrópodes Vetores (Laficave), Instituto Oswaldo Cruz (IOC), Fundacao Oswaldo Cruz (FIOCRUZ), Rio de Janeiro CEP 21040-360, Brazil

**Keywords:** mosquito-borne diseases, arboviruses, mosquito surveillance, insecticide resistance, France

## Abstract

**Simple Summary:**

To date, mosquito resistance to insecticides highly jeopardizes all the efforts made worldwide in vector control. In this context, an integrated plan of resistance surveillance is proposed to respond gradually and specifically to the various situations that may be encountered in the field. Initially developed for France (Metropolitan and the French Overseas territories), the plan can be easily adapted to other countries because it is rooted in the use of WHO standardized test methods and indicators for resistance monitoring and the rules for evaluating the Risk of Resistance (RIR) at a territory level can be used whatever the entomological and/or epidemiological situation. The integrated plan is expected to provide countries with technical guidance on how and when to implement surveillance of insecticide monitoring in mosquitoes of public health importance.

**Abstract:**

Mosquito-borne diseases such as malaria, dengue, or chikungunya have been re-emerging all over the world, including in Europe. Managing resistance to public health pesticides in mosquitoes is essential and requires global, integrated, and coordinated actions and strong engagement of decision-makers, scientists, and public health operators. In this context, the present work aims at proposing an integrated plan of resistance surveillance in France and in the French Overseas territories in order to provide graduated and appropriate responses according to the situation. Briefly, the plan relies on periodic monitoring of insecticide resistance at the population level in predefined sites using adequate biological, molecular, and/or biochemical approaches and a stratification of the level of resistance risk at the scale of territory to adjust surveillance and vector control actions. The plan relies on the latest methods and indicators used for resistance monitoring as recommended by the World Health Organization in order to prevent or slow down its extension in space and time. The plan has been developed for France but can be easily adapted to other countries in order to provide a coordinated response to the growing problem of mosquito resistance in Europe.

## 1. Introduction

Rapid climate changes and the intensification of human activities on a global scale, especially trading, increase the risks of the introduction of invasive species and modify the geographic distribution of those already present [1]. It is also the case of species vectors of human, animal, and plant diseases [2]. It is, therefore, foreseeable that vector control actions against these species will intensify and favor the emergence and spread of insecticide resistance [3]. This is already the case for species such as *Aedes aegypti* and *Anopheles gambiae* in many parts of the world. Thus, for example, resistance to insecticides in *Anopheles* was found in 78 countries with ongoing malaria transmission [4], while resistance to *Aedes* spp. was found in at least 57 countries at risk of *Aedes* borne disease transmission [5].

Resistance to pyrethroid insecticides is high and frequent in *Ae. aegypti* and is rapidly increasing in *Ae. albopictus* in several regions of the world, such as the Indian Ocean or Europe, with the increase in insecticide treatments in response to the recurrent epidemics of arboviral diseases, such as Chikungunya or Zika [6,7,8].

If Metropolitan France is still relatively preserved, the French overseas territories (Martinique, Guadeloupe, French Guiana, La Reunion, Mayotte, New Caledonia, and French Polynesia) are already strongly affected by insecticide resistance in mosquitoes, especially those responsible for the transmission of arboviruses.

Indeed, France has a high geographical and administrative diversity. This leads to a large diversity of vector species that are found with different levels and frequencies of resistance in space and time [9]. Global warming is also a factor that may affect the distribution, vectorial capacity, and insecticide susceptibility of mosquitoes that are vectors of pathogens [10,11]. Moreover, the French territories are faced with very different epidemiological situations, ranging from the absence of human diseases transmitted by mosquitoes (e.g., Northern France) to sporadic transmission (e.g., dengue and chikungunya in Southern France), seasonal (e.g., malaria in French Guiana), annual (e.g., dengue in Martinique or French Guiana), and/or epidemic (e.g., chikungunya in La Reunion Island), or even endemic (e.g., dengue in La Reunion Island). Last, the proximity of these territories to other countries where insecticide resistance is already present (e.g., Indian Ocean, South America, Caribbean) may result in the introduction of resistance alleles to France and subsequently to all European territories.

In this context, the present work aims at proposing an integrated plan of resistance surveillance in the whole French territory to guarantee a fast and adapted response in case of identification and diffusion of resistance in order to maintain the efficiency of vector-borne disease control. The plan could be adapted to other countries in order to provide a timely and locally adapted response to the growing problem of mosquito resistance in Europe. 

The general principle of this plan of surveillance of insecticide resistance (PSIR) is the early detection of resistance to insecticides in current use and/or usable at the scale of a territory and to provide graduated and appropriate responses according to the situation. This plan relies on the updated methods and indicators for resistance surveillance recommended by the World Health Organization (WHO) [12,13,14] and the WIN international network [15,16] in order to prevent or slow down its extension in space and time.

The integrated PSIR is characterized by actions to be taken at two levels:

- Periodic monitoring of insecticide resistance at the population level. This implies the routine surveillance of the levels and mechanisms of resistance in predefined sites using biological, molecular, and/or biochemical approaches.
- Stratification of the level of Risk of Resistance (RiR) at the scale of a territory to assist decision-making: This involves estimating the level of risk of insecticide resistance in this territory based on population surveillance data in order to adjust surveillance and vector control actions.

The proposed approach also takes into account the regulatory, epidemiological, and geographical particularities of the French territories, especially the temporary use of molecules by way of derogation in exceptional circumstances (e.g., strong resistance and epidemic situation) and when no other alternatives exist.

## 2. Surveillance of Insecticide Resistance at the Population Level

This surveillance should be performed periodically in the same sites in order to evaluate the baseline susceptibility level of resistance and detect any changes in resistance level and frequency over time.

Resistance tests should primarily focus on the insecticides used in vector control in the given territory, such as deltamethrin and *Bacillus thuringiensis israelensis* (Bti) in France, for biological resistance tests on adults and larvae, respectively. However, an extension of monitoring activities to other insecticides used in vector control and to molecules that can be used by way of derogation should be planned in the case of incipient resistance to the commonly used insecticides [17].

### 2.1. Methodology for Monitoring Insecticide Resistance within a Territory

#### 2.1.1. Selection of Sentinel Sites

For effective monitoring of insecticide resistance, surveillance must be conducted through sentinel sites that have been pre-selected for their ability to represent the range of vector species of epidemiological interest and the eco-epidemiological and geographical areas of the territory of concern. The setting of such a panel of sentinel sites aims to early detect the emergence of resistance and to follow its evolution in space and time in a territory of concern.

The criteria mostly used for the selection of sentinel sites are as follows.

- Level of epidemiological risk (e.g., areas with a high risk of transmission of arboviruses and/or malaria, border areas).
- Frequency and the number of insecticides used for both public health and agriculture (e.g., irrigation areas colonized by malaria vectors; urban and peri-urban areas for *Aedes* and *Culex*).
- Human population density (urban versus rural).
- Entomological risk level (presence and abundance of vectors through entomological surveillance).
- Accessibility/specificity of the sentinel sites.

#### 2.1.2. Frequency of Monitoring

In many countries, there is no coordinated and sustained surveillance of insecticide resistance in mosquitoes. Resistance monitoring is sometimes performed “empirically” in response to outbreak events and/or vector control failures. To detect resistance in natural populations at an early stage and to respond quickly to the need, it is recommended to collect data (biological and molecular) over several years under the same conditions. Thus, it is recommended to test the susceptibility of mosquitoes to the insecticides used in vector control at least once a year in each sentinel site [18]. This should be performed preferentially during the period of abundance of the local vector populations and/or just before the peak of transmission.

#### 2.1.3. Collection Methods and Sampling

Numerous methods are used to collect mosquitoes for resistance testing, both in the immature state (larvae and eggs) and as adults [19]. 

The goal is to be able to propose a large network of capture/collection sites that are attractive to the target species and do not change over time and space. This allows the collection of a maximum number of individuals for the sensitivity tests.

Regarding *Aedes* spp. and *Culex* spp., prospecting in larval sites is recommended because this allows us to quickly collect a large number of larvae within a limited space. For the *Anopheles*, it is often more difficult to locate the larval sites (except the rice-growing species) and/or to have productive sites. Consequently, the collection of adult mosquitoes is usually carried out by using backpack vacuums and/or baited traps. 

Whatever the capture method used, it is important to prospect at least ten locations per sentinel site in order to maximize the representativeness of the sampling. Indeed, it is crucial that the samples to be tested are taken from several sampling sites spaced apart to reduce the probability of collecting related individuals.

- Recommendations during larval surveys and/or when using oviposition traps;

In the absence of specific recommendations on the collection of samples for resistance monitoring, and based on the recommendations of the European Centre for Disease Prevention and Control (https://www.ecdc.europa.eu/en) (accessed on 9 February 2023) on the surveillance of invasive mosquitoes in Europe (2012), we recommend the following methods, depending on the target species and for each sentinel site:

  - Setting at least four oviposition traps/ha over a period of 4 days and/or,
  - Prospecting at least 10 breeding sites with a distance of at least 100 m between capture sites.

When prospecting, it is important to note the type of breeding site (e.g., oviposition trap, barrel) and the GPS coordinates, as some sites may be polluted. Prospecting should not be carried out just after a recent vector control or agricultural treatment (e.g., 2 weeks is equivalent to 1–3 generations of mosquitoes). Samples collected within the same site may be pooled in order to have a sufficient number of mosquitoes for biological and/or molecular testing (see Table S3 in the Supplementary Materials).

- Recommendations when catching adult mosquitoes;

Collections are generally made by trapping (e.g., light), living baits that lead to ethical problems, the use of chemicals (CO_2_, attractants…), and/or backpack hoovers (e.g., Prokopack^TM^ hoover or Insectavac^TM^). Samples should be sorted and stored during transport (air-conditioned if possible) and then taken back to the laboratory for identification. In practice, WHO [13] recommends performing biological tests on F1 offspring obtained from at least 30 wild-caught females.

### 2.2. Sample Selection for Biological Tests

The age, physiological state, and gender of the mosquitoes can influence the test results [13]. The use of males is not recommended as they are more sensitive to insecticides and are more fragile than females (higher mortality in controls). The tests are therefore carried out only on adult females aged 3 to 5 days, non-blood fed, and not exposed to xenobiotics.

In order to obtain standardized results according to the stage and/or age of the adults, it is recommended that the susceptibility tests be carried out on the progeny of wild-caught mosquitoes, commonly referred to as the F1 generation reared under standardized conditions (larval density, food, temperature, relative humidity, etc.). In case the F1 generation cannot be used (e.g., absence of an insectarium), it is possible to perform the tests directly on wild-caught females (F0). The advantages and disadvantages of using wild females (F0) and/or their offspring (F1) are given in Table 1.

### 2.3. Selecting Biological Tests for Resistance Surveillance

The choice of the bioassay to be used for testing resistance will depend on the insecticides of concern (e.g., larvicides vs. adulticides) and the availability of diagnostic concentrations for each insecticide family and for a given vector species.

The recommended biological methods (bioassays) for testing the susceptibility of populations to insecticides in sentinel sites are the following:

- The larval bioassay (or larval test) allows testing the resistance of mosquito populations to a larvicide [12]. The method consists of exposing larvae from a given population (in parallel to the susceptible reference strain) to increasing concentrations of a larvicide to estimate the Lethal Concentrations (LCs) and thus calculate the Resistance Ratio 50 or RR50 [12]. In the case of insect growth regulators (IGRs), the effect of the insecticide on mosquito larvae is expressed as the percentage of larvae that do not emerge as adults compared to the control and is therefore referred to as inhibition of adult emergence [12].

- The WHO tube test method [20] allows testing the resistance of adult mosquito populations to diagnostic concentrations (or discriminating concentrations) of insecticides (Tables S1 and S2 in the Supplementary material). The method relies on exposing mosquitoes to filter papers impregnated with an insecticide for assessing the resistance status of a given population [13]. If resistance is observed or suspected, it is recommended to perform intensity tests by exposing mosquitoes to 5 and 10 times the WHO recommended diagnostic concentration in order to evaluate the level of resistance (low, moderate, or high). The standard operating procedure of the WHO tube test is available at: https://www.who.int/teams/global-malaria-programme/prevention/vector-control/insecticide-resistance (accessed on 2 February 2023).
- The WHO bottle test is another method recently developed by the WHO to assess the resistance of adult mosquito populations to insecticide molecules that cannot be impregnated on filter paper due to crystallization [13,14,21]. This test is based on the impregnation of glass bottles with a diagnostic concentration in order to assess the susceptibility status of the studied population to an insecticide (Tables S1 and S2 in the Supplementary material). However, there are no contraindications to the possibility of doing intensity bioassays with the bottles; the WHO still has not validated the use of tests with 5 and 10 times the diagnostic concentration for the recently evaluated molecules. This method also allows for performing dose-response tests when the diagnostic concentrations are not available. The standard operating procedure of the WHO tube test is available at: https://www.who.int/teams/global-malaria-programme/prevention/vector-control/insecticide-resistance (accessed on 9 February 2023). The SOP for testing adult mosquito resistance to IGRs (e.g., pyriproxyfen) in the WHO bottle assays is available at https://www.who.int/publications/i/item/9789240043794 (accessed on 9 February 2023).

The list of diagnostic concentrations recommended by WHO for different insecticides and species can be found in the Supplementary material (Tables S1 and S2).

The recommended number of mosquitoes to be used in the bioassays is given in Table S3 (Supplementary material).

Biological tests on mosquito populations are usually carried out only once at a given time. Where it is not possible to test the minimum number of mosquitoes in a single day, testing can be performed over several days until this number is reached if control tests are performed in parallel. For adult tests, impregnated papers can remain in the tubes if they are wrapped in aluminum and kept at 4 °C between two tests. For the bottle tests, there are currently no recommendations on the number of uses and/or the storage time of bottles impregnated with the WHO diagnostic concentrations. It is, therefore, preferable to reprocess the bottles if new tests have to be performed.

### 2.4. Indicators of Resistance

The main indicator measured in susceptibility testing is the mortality of mosquitoes after exposure to a diagnostic concentration or to increasing concentrations of insecticide. Mortality is determined 24 h after exposure with some exceptions (e.g., chlorfenapyr, for which mortality is recorded at 72 h post-exposure). Mortality in the control should always be measured in parallel, if necessary, used to correct the mortality of treated mosquitoes [22]. As regards the IGRs, the indicator will be the adult emergence inhibition rate (i.e., EI%) for larval bioassays [12] and the inhibition of female oviposition for adult bioassays [14].

### 2.5. Interpretation of Biological Tests

#### 2.5.1. Larval Test

Mortality data at each tested concentration are used to estimate the lethal concentration for 50% of the population (LC50) or the concentration inhibiting 50% of adult emergence (IE50) by log-probit regression [23,24].

**Figure 1:**
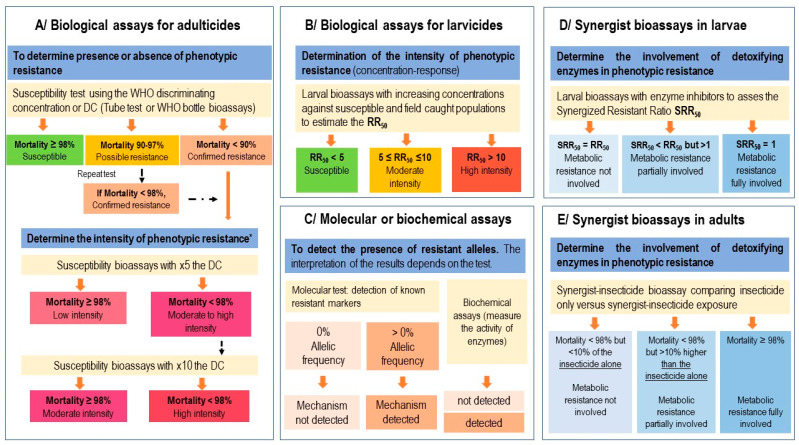
Summary diagram of resistance surveillance in adult and larval mosquitoes (modified from WHO [13]).

The RR50 value is obtained by comparing the LC50 (or IE50) of the tested population versus the susceptible reference strain. The RR50 provides an estimate of the strength of resistance of a mosquito population to a larvicide and serves as a basis to estimate the phenotype of resistance (Figure 1).

#### 2.5.2. Adult Test

These tests measure the mortality rate of a given population at the diagnostic concentration (DC) according to standardized protocols [13,14] and, therefore, the proportion of resistant individuals in a given sentinel site. The WHO criteria of susceptibility and resistance after mosquito exposure to a DC or to 5 and 10 times the concentration is summarized in Figure 1.

### 2.6. Use of Molecular and/or Biochemical Tests

In parallel with biological tests, it is also recommended to perform molecular and possibly biochemical tests to detect the presence of the most likely resistance alleles and to estimate their current frequency in natural populations. This can be performed on a subsample of mosquitoes previously used in susceptibility testing but preferably by using mosquitoes that were not previously exposed to any insecticides (control batches). This is particularly important in order to (i) detect the emergence of resistance at an early stage, (ii) identify the mechanisms involved and anticipate possible cross-resistance (i.e., resistance to more than one insecticide family), and (iii) anticipate possible operational failures. This information will be used for planning and implementing vector control and surveillance operations, in particular, to guide the choice of insecticides to be used in the field. Finally, estimating the frequency of the alleles of resistance is essential for evaluating the effectiveness of a resistance management program (e.g., mosaics, rotations, insecticide mixtures) [25].

Because there are no international recommendations on molecular resistance detection methods (as there are for biological tests), we cannot recommend reference methods to apply in the PSIR framework. However, many biochemical and molecular tools can be used to detect the presence of resistance markers [26]. Whatever the method used, it is suggested that at least 50 individuals per sentinel site should be tested for a resistance marker present at an allelic frequency of 1% (2 N chromosomes).

Considering the lack of standardization with regard to molecular diagnostic assays, harmonization of test protocols by national reference centers could be an option to regularly update the list of relevant resistance markers to be monitored in the field.

### 2.7. Use of Synergistic Tests (Enzyme Inhibitors)

In the absence of technical and/or logistical capacity to perform molecular and/or biochemical tests, the use of synergistic tests can identify the detoxification enzymes involved in the resistance (metabolic resistance). The detailed protocol for synergistic testing is described in WHO [13], and the standard operating procedures are available at: https://www.who.int/publications/i/item/9789240043855 (accessed on 9 February 2023).

In adults, synergist testing is only recommended if mortality rates of mosquitoes exposed to diagnostic concentrations are <90% (as the effect of the synergist can only be reliably assessed if mosquito resistance is confirmed). In the end, it is possible to determine whether the phenotype of resistance is not partially or totally due to metabolic resistance by detoxifying enzymes.

The interpretation of the results according to the WHO [13] criteria is as follows.

- The insecticide sensitivity is fully restored after pre-exposure to the synergist molecule (i.e., mortality ≥ 98% in samples exposed to the synergist/insecticide combination). This suggests that a metabolic resistance mechanism is solely responsible for the observed resistance phenotype.
- The insecticide sensitivity is partially restored after pre-exposure to the synergist molecule (i.e., mortality in samples exposed to the synergist/insecticide combination < 98% but 10% higher than mortality with the insecticide alone). This suggests that a mechanism involving detoxification enzymes is partly responsible for the observed resistance phenotype but that other resistance mechanisms also contribute to it.
- The insecticide sensitivity is not restored after pre-exposure to the synergist molecule (i.e., mortality in samples exposed to the synergist/insecticide combination < 98% but not more than 10% higher than mortality with the insecticide alone). This suggests that the observed resistance phenotype does not involve detoxification enzymes.

The overall outline of resistance surveillance in adult and larval mosquitoes is shown in Figure 1. 

## 3. Stratification of Resistance Risk on a Territorial Scale and Decision Support

Resistance is a dynamic phenomenon in space and time under the influence of gene flow between mosquito populations, demographic effects, and also variations in selection pressures [16,27]. The assessment of the level of risk should not be limited to the “population” level but should also take into account the diversity of situations on the territory scale in order to propose suitable vector control and resistance management actions in a given geographical area. Thus, guidelines have to be interpreted and tuned according to the local context. Indeed, depending on geographical, administrative, and epidemiological diversity, some criteria may not be applicable to all cases. Vector control stakeholders, in conjunction with decision makers, are best placed on defining with coherency the strategies to be adopted for the territory of concern.

### 3.1. Definition of the Territory

The concept of territory is complex to define with accuracy. This definition can vary according to local constraints (geographical and/or administrative) but also according to the vector species monitored. Overall, a territory should be defined by (i) its geographical and ecological homogeneity, (ii) the active and passive dispersion capacities of the vector species monitored, and (iii) its homogeneity in terms of surveillance and vector control activities. A territory can therefore be a geographical area or an administrative entity such as a local community, a department, or a region. In the PSIR, the territory is, therefore, an entity on the scale to which a homogeneous and well-suited resistance management strategy is applicable.

In this context, the territory must be defined beforehand by the surveillance, as the number and distribution of sentinel sites depend on it. The number of sentinel sites in a territory is an essential parameter for effective monitoring of dynamics of resistance in space and time. The greater the number of sentinel sites, the more accurate the surveillance will be. Obviously, this number also depends on the surface area of the territory to be surveilled, its ecological and epidemiological heterogeneity, and the human and financial resources available. However, it is recommended to have at least 10 sites for a given territory.

### 3.2. Stratification of the Mosquito Resistance Risk

In order to facilitate the classification and comparison between territories, four levels of Risk of Resistance (RiR) have been defined. The RiR depends on two criteria (i) the levels of resistance observed at the population levels (as given in Table 2) and (ii) the proportion of resistant populations at the territory level (Table 3). A RiR is assessed for a vector species-active substance pair in a given territory. For determining the RiR level, it is necessary to consider the frequency of sentinel sites with the highest levels of resistance and then refer to Table 3.

Each RiR level implies actions in terms of surveillance or vector control.

- Resistance risk 0 (RiR 0)

A RiR score of 0 indicates that the territory is considered to be at low risk of resistance to the insecticide tested. This ranking is given to a territory if no resistance has been detected. This means an adult mortality ≥ 98% at WHO diagnostic concentrations or RR50 < five for larvae in all sentinel sites or less than 10% of the sentinel sites monitored have a level of resistance equal to 1. Routine resistance monitoring should be maintained.

- Resistance risk 1 (RiR 1)

A RiR score of one indicates a situation where resistance is beginning to emerge in relation to the insecticide tested and corresponds to a situation where resistance is reaching a frequency close to the “tipping point” [25]. This corresponds to a point where resistance alleles are present at a low frequency but are likely to increase extremely rapidly if no actions are undertaken (e.g., a resistance allele that is present at 2% and doubles at each generation will, in theory, be fixed in only six generations). RiR 2 should therefore lead to an adaptation of the monitoring system at the populational level. This classification is attributed to a territory if >10% of the sentinel sites show a resistance level 1 or if isolated populations show a resistance level 2 in the territory.

- Resistance risk 2 (RiR 2)

A RiR score of two indicates a moderate resistance (adult mortality < 98% at five times the WHO diagnostic concentrations and ≥ 98% at 10 times WHO diagnostic concentrations or 5 ≤ RR50 ≤ 10 for larvae) widespread throughout the territory or the presence of a few isolated populations showing strong resistance to the insecticide tested. In this situation, there is a risk of decreased efficacy of a given insecticide at the territory level or a risk of vector control failure locally for that insecticide. This score is attributed to a territory when more than 10% of the sentinel sites are at resistance level 2. It is also attributed as soon as the first detection of a level of resistance 3 in a site is observed and as long as this does not exceed a proportion of 50% in the territory.

- Resistance risk 3 (RiR 3)

A RiR score of three indicates a strong resistance (adult mortality < 98% at 10 times WHO diagnostic concentrations and/or RR50 > 10 for larvae) that is widespread to the territory on which the insecticide is used. There is a high risk of a total loss of control of the vector species in the territory of concern. This scoring is given to a territory when more than 50% of the sentinel sites are at resistance level 3.

The thresholds for the population proportions at the territory level are given for general guidance purposes only. These thresholds can be adjusted according to the vector species of concern, the configuration of the territory, or the distribution of sentinel sites.

Examples of resistance risk stratification for some French territories are given in Appendix A (Table A1, Table A2, Table A3, Table A4 and Table A5).

### 3.3. Decision Support

The allocation of territory in one of the RiR categories should lead to quick action to stop the spread of resistance. The aim is to preserve the lifespan of the insecticides and to avoid any operational failure. The actions to be refined according to the RiR are of two types: on the one hand, actions related to resistance monitoring and, on the other hand, vector control actions (Table 4).

Resistance surveillance should be strengthened from RiR 1. The transition to RiR 2 should lead to the selection of alternative active substances that are authorized and/or can be used on a derogatory basis, and biological tests should be performed to assess their potential for resistance management and for use in vector control.

The characterization of resistance mechanisms and their respective frequencies in populations is also recommended, as this provides information that can have an impact in terms of management (cross-resistance, possible reversion of resistance, spatio-temporal dynamics, etc.).

Actions in terms of surveillance must be accompanied by modifications of vector control practices in order to stop an unfavorable evolution of resistance in the territory concerned or even to reverse its dynamics when possible. This can be performed by reducing the frequency of treatments, alternating authorized insecticides in time or space, or favoring non-chemical control actions. Finally, the transition to RiR 2 is accompanied by (i) the selection of alternative active substances that are authorized and/or can be used on a derogatory basis and (ii) the performance of biological tests as well as efficacy tests of alternative substances under semi-operational conditions in order to anticipate the change of substance used in vector control.

In RiR 3, an important change in vector control practices is expected. The interruption of the use of the insecticide of concern, as well as those with cross-resistance, on the target species is recommended in the territory because its operational efficacy is no longer guaranteed or even problematic because it can lead to the fixation of resistance alleles. Complementary control methods, namely physical, biological, and genetic, should be introduced, even preferred if pilot studies have been conducted in the given territory (e.g., the release of sterile male mosquitoes) and if accompanied by rigorous monitoring and evaluation of their efficacy and acceptability. Awareness-raising campaigns among political stakeholders, civil society, and communities and their involvement in vector control actions are also important factors for a successful control, especially when new control tools are used [28].

Finally, the fact that resistance risk management is assessed at the territorial level should not prevent targeted actions at the local level. Thus, the detection of strong resistance in a sentinel site (resistance level 3) should, at least, lead to increased attention during future surveillance and, if possible, to the cessation of the use of the insecticide concerned in the area.

## 4. Usable Substances Based on Derogatory Procedures

The use of non-authorized molecules for vector control could be necessary for the event of a high risk of resistance in a territory and the occurrence of an outbreak (e.g., dengue or chikungunya epidemic). To date, there are active substances that can potentially be used in vector control [17,29,30,31]. However, due to the diversity of the resistance mechanisms, it is crucial to empirically assess the potential for cross-resistance between these molecules and those already impacted by resistance in the field. In addition, resistance mechanisms to biocides that are no longer authorized can sometimes persist in populations [32].

It is therefore recommended to identify active substances that can potentially be used according to derogatory procedures as early as RiR 2 in order to avoid having to use alternative substances for which the vectors are already resistant in the field. The use of these molecules in vector control is only recommended at the RiR 3 level. Finally, in the context of the identification and selection of active substances for derogatory use, several criteria need to be taken into account, such as the deadline to establish the list of substances and authorization to use them, the availability of a test protocol as well as historical data on resistance to these molecules in other countries and/or other species (literature monitoring). The use of non-authorized molecules for vector control could be necessary in the event of a high risk of resistance in a territory and the occurrence of an outbreak (e.g., dengue or chikungunya epidemic). 

It is, therefore, recommended to identify active substances that can potentially be used according to derogatory procedures as early as RiR 2 in order to avoid having to use alternative substances for which the vectors are already resistant in the field. The use of these molecules in vector control is only recommended at the RiR 3 level. Finally, in the context of the identification and selection of active substances for derogatory use, several criteria need to be taken into account, such as the deadline to establish the list of substances and authorization to use them, the availability of a test protocol as well as historical data on resistance to these molecules in other countries and/or other species (literature monitoring).

## 5. Potential Contributors and Actors

In order to implement an integrated approach for insecticide resistance surveillance, it will be essential to involve all the different stakeholders involved in mosquito control and surveillance in a territory such as:

- A regional health authority (ARS (French Regional Health Agency) in France) will be responsible for steering and implementing the PSIR plan. This organization will be in charge of setting up an agreement with the competent partners (operators, local authorities, research laboratories) to ensure the implementation of the resistance surveillance plan in the sentinel sites (before implementing the monitoring, it is necessary to define the size of the territory, the number of sentinel sites to be monitored, the frequency of the monitoring, the number of insecticides and the type of tests to be carried out according to the species).
- Field operators who will supervise field surveys and collect data/samples in the identified sentinel sites. Depending on their training and access to adequate infrastructure, these operators may also be involved in biological testing (local authorities, private sector, etc.). They will ensure a permanent link with the central authority in charge of the surveillance.
- Specialized laboratories (private companies and/or academic research laboratories) that can provide technical assistance in setting up monitoring protocols (biological, molecular, and biochemical test methods), implementing new control strategies (laboratory and operational tests), and analyzing and interpreting data.

The development of an integrated surveillance plan will require:

- A local steering to delineate the perimeter of the territory and describe the objectives and context of the surveillance as well as the tools to do it correctly.
- A description of the operation and management of resources, data processing and interpretation, and monitoring methods (event-based monitoring/programmed monitoring),
- A link with the training and communication around the plan of surveillance.
- A regional coherency and national coordination. Indeed, a network organization has the advantage of rapidly sharing experiences, knowledge, and sometimes tools. This allows for saving time and resources.

All the collected data could then be grouped and analyzed within a National Observatory of Mosquito Resistance responsible for ensuring the proper application of protocols, data analysis, and the creation of resistance threat maps on a national scale in order to provide a timely, appropriate and graduated response according to the risk.

## 6. Conclusions

Insecticide resistance in mosquito vectors of human diseases is growing worldwide, including in Europe. Resistance is considered by the WHO as a major threat to the control of mosquito-borne diseases and has likely contributed to the reemergence and/or to the spread of several arboviruses worldwide [15]. Urgent actions are required to prevent resistance spread and to maintain the effectiveness of vector control interventions in the short, medium, and long term [33].

A decade ago, WHO developed the Global Plan for Insecticide Resistance Management (GPIRM) in mosquitoes [25] to trigger coordinated action from all stakeholders for integrated practices for managing insecticide resistance in countries at risk of mosquito-borne diseases and to preserve the efficacy of the current vector control methods. Unfortunately, since 2012, very few countries have effectively implemented the GPIRM, mainly because of insufficient technical guidance on how and when to implement resistance surveillance and management [33].

In this context, guidelines for the implementation of a PSIR applicable to all French territories are proposed. These guidelines are also intended to guide the surveillance plans of other countries. This plan is based on the regular monitoring of the levels and mechanisms of resistance at the population level in sentinel sites and, on the other hand, on an estimate of the level of risk of resistance at the territory level (RiR) aimed at guiding the actions to be carried out in terms of surveillance and vector control.

The PSIR is mainly rooted in the WHO recommendations dealing with testing, sampling practices, indicators, and data interpretation. Proper sizing of the human and financial resources in each territory is a key element to consider for its setting.

While in some French territories, the mechanisms of resistance are identified and monitored, for others, the monitoring actions are “incomplete” or even lacking and should be strengthened and sustained, both from the point of view of the organization of the surveillance plan and the continuity of monitoring actions.

For territories already subject to a high RiR level (e.g., Martinique, Guadeloupe, French Guiana; See Appendix A: Table A1, Table A2, Table A3, Table A4 and Table A5), it is advisable to propose changes in the practices based on innovative and environmentally friendly techniques that may include physicochemical (e.g., attractant traps, eave tubes, autodissemination), biological (e.g., predators, entomopathogenic bacteria) and/or genetic (e.g., SIT,) control actions whose efficacy has been demonstrated. These alternative strategies, in addition to their public health impact, could mitigate insecticide resistance in mosquito vectors due to their specific mode of action in insects (see review in Achee et al. [34]).

This evolution of practices will have to be adopted by the operators and supported by scientific, health, ethical, and political bodies to envision actions whose social and environmental acceptability will allow their implementation.

In the meantime, the active substances that can be used in vector control, even more than in agriculture, must be considered a limited resource. Their reduced number makes them a key issue in the control of vector species and a common good whose efficacy must be preserved, just like antibiotics [35,36,37]. The evolution of resistance to these biocides is, therefore, a worrying issue that requires particular attention. This evolution in response to selection pressure is a dynamic phenomenon over time. This is why effective monitoring has to be conceived in the long term and in a coordinated manner by territory.

The following recommendations are proposed to improve resistance surveillance at the French and European levels:

- Include resistance surveillance as one of the duties of vector control.
- Implement the PSIR in all French and European territories because mosquitoes recognize no barriers or borders.
- Promote applied and fundamental research projects allowing a better knowledge of the dynamics of resistance, its management and its impact on vector control, the identification of new insecticides and synergists, and the development of new enviro-friendly and socially accepted vector control strategies.
- Creation of a national (even European) observatory of resistance to coordinate the monitoring plans conducted in each territory.
- Assess the efficacy of new vector control approaches (autodissemination, trapping, sterile insect technique).
- Promote insecticide-free methods for sustainable vector control that is acceptable to the population.

## Figures and Tables

**Table 1:** Advantages and disadvantages of using F1 generation (progeny) or F0 generation (wild females) for resistance testing (modified from [13]).

Generation	Advantages	Drawbacks
F1 generation (progeny)	Age of the vectors is known during the tests, which allows direct comparison of the results. Allows having a sufficient number of mosquitoes for testing.	Requires adequate infrastructure (laboratories and insectarium), which limits the locations where testing can be performed. Low diversity and representativeness of samples if the sample size is small.
F0 generation (wild mosquitoes)	Requires few facilities because tests can be performed directly in the field. Allows for more accurate and direct quantification of the frequency of resistance alleles in natural populations.	The age and physiological status of the vectors are unknown, which increases the variability of the tests and limits the comparison of results. Mosquitoes may have been pre-exposed to an insecticide/xenobiotic, thereby altering test results. Wild-caught females are potentially infected and should be handled with care.

**Table 2:** Indicators used to define the level of resistance at the population level.

Biological Tests (WHO) ^1^	Resistance Status (Biological Tests)	Resistance Mechanisms ^2^		Resistance Level
		Molecular and/or Biochemical Markers	Enzymatic Inhibitors (Synergist Test)	
≥98% mortality at WHO DC (adults) or RR50 < 5 (larvae)	Susceptible	Absent (or not tested)		0
	Susceptible but presence of resistance alleles	Present at low frequency (=Tipping point)		1
<98% mortality at 5× the WHO DC but ≥98% mortality at 10× the WHO DC (adults) or 5 ≤ RR50 ≤ 10 (larvae)	Moderate resistance	Present (or not tested)		2
<98% mortality at 10× the WHO DC (adults) or RR50 > 10 (larvae)	Strong resistance	Present (or not tested)		3

DC: Discriminating Concentration; RR_50_: Resistance Ratio 50. ^1^ Biological adult tests with discriminating concentrations defined by the WHO (WHO DC) or on larvae (dose-response) provide the indicators required to assess resistance at the population level. ^2^ Presence of resistance alleles based on molecular tests (known resistance markers), biochemical tests (enzymatic activities) or biological tests with enzymatic inhibitors (effect of synergists on the resistance phenotype). These indicators are complementary to biological tests and allow a more detailed characterization of resistance mechanisms in a population.

**Table 3:** Stratification of resistance risk at the territory level.

		Proportion of Sites across the Territory		
		Isolated Sites (<10%)	Multiple Sites (10–50%)	Majority of Sites (>50%)
Level of resistance at the population level (Table 2)	0	RiR 0	RiR 0	RiR 0
	1	RiR 0	RiR 1	RiR 1
	2	RiR 1	RiR 2	RiR 2
	3	RiR 2	RiR 2	RiR 3

**Table 4:** Recommended surveillance and vector control actions according to the RiR score at the territory level (for a given insecticide and species).

RiR	Resistance Monitoring	Vector Control Actions (VCAs)
RiR 0	Routine monitoring to be maintained.	VCA unchanged.
RiR 1	Strengthening of resistance monitoring (increase in the number of sites and frequency of sampling).	VCA unchanged. Conceptualization of a locally adapted resistance management plan.
RiR 2	Continued monitoring of resistance. Selection and integration of alternative molecules (authorized and/or usable on a derogatory basis) in the monitoring. Carrying out targeted sensitivity tests on the populations showing the strongest resistance to evaluate their potential in resistance management.	Moderate modifications of VCA: reduction of treatments, alternation of authorized insecticides in space/time, use of complementary methods (trapping, physical control, etc.). Efficiency trials under semi-operational conditions with alternative molecules authorized and/or usable on a derogatory basis.
RiR 3	Continued monitoring of resistance. Integration of alternative molecules (authorized and/or usable on a derogatory basis) in the territorial monitoring.	Significant changes in VCA: cessation of treatment, use of alternative insecticides that are authorized and/or that can be used on a derogatory basis (e.g., epidemic), of innovative control strategies (if a proof of concept exists), etc.

## Data Availability

Data are contained within the article or supplementary material. No new data have been generated in this study. Only publicly available datasets were analyzed.

## Supplementary Materials

The following supporting information can be downloaded at: https://www.mdpi.com/article/10.3390/insects14050457/s1, Table S1: Insecticide discriminating concentrations for WHO susceptibility bioassays with *Anopheles* mosquitoes [13,14,38,39,40,41]: Table S2: Insecticide discriminating concentrations for WHO susceptibility bioassays with *Aedes* mosquitoes [13,14]; Table S3. Recommended number of mosquitoes to use in the resistance surveillance tests.

## Appendix A

**Table A1:** RiR in French overseas territories with regards to resistance of *Culex quinquefasciatus* to deltamethrin.

		Proportion of Sites across the Territory		
		Isolated Sites (<10%)	Multiple Sites (10–50%)	Majority of Sites (>50%)
Level of resistance	1	RiR 0 French Polynesia	RiR 1 La Reunion	RiR 1 Mayotte
	2	RiR 1	RiR 2	RiR 2
	3	RiR 2	RiR 2	RiR 3 Guadeloupe, Saint-Martin, Martinique, French Guiana

**Table A2:** RiR in French overseas territories with regards to resistance of *Aedes aegypti* to deltamethrin.

		Proportion of Sites across the Territory		
		Isolated Sites (<10%)	Multiple Sites (10–50%)	Majority of Sites (>50%)
Level of resistance	1	RiR 0 Mayotte, French Polynesia, La Reunion	RiR 1	RiR 1
	2	RiR 1	RiR 2	RiR 2 Martinique
	3	RiR 2 New Caledonia	RiR 2	RiR 3 Guadeloupe, Saint-Martin, French Guiana

**Table A3:** RiR in Metropolitan France, Corsica, Mayotte and La Reunion with regards to resistance of *Aedes albopictus* to deltamethrin.

		Proportion of Sites across the Territory		
		Isolated Sites (<10%)	Multiple Sites (10–50%)	Majority of Sites (>50%)
Level of resistance	1	RiR 0 Metropolitan France, Corsica, Mayotte	RiR 1	RiR 1 La Reunion
	2	RiR 1	RiR 2	RiR 2
	3	RiR 2	RiR 2	RiR 3

**Table A4:** RiR in some French overseas territories with regards to resistance of *Aedes aegypti* to temephos.

		Proportion of Sites across the Territory		
		Isolated Sites (<10%)	Multiple Sites (10–50%)	Majority of Sites (>50%)
Level of resistance	1	RiR 0 Mayotte, French Polynesia	RiR 1	RiR 1 La Reunion
	2	RiR 1	RiR 2	RiR 2
	3	RiR 2	RiR 2	RiR 3 Martinique, Guadeloupe

**Table A5:** RiR in some French overseas territories with regards to resistance of *Aedes aegypti* to malathion.

		Proportion of Sites across the Territory		
		Isolated Sites (<10%)	Multiple Sites (10–50%)	Majority of Sites (>50%)
Level of resistance	1	RiR 0 New Caledonia, French Polynesia	RiR 1	RiR 1 Guadeloupe, French Guiana
	2	RiR 1	RiR 2	RiR 2
	3	RiR 2	RiR 2	RiR 3
