# Supplementary material for: Integrated Plan of Insecticide Resistance Surveillance in Mosquito Vectors in France
Source: Insects. 2023 May 12;14(5):457. doi: 10.3390/insects14050457 (PMC10231048; doi:10.3390/insects14050457)
Supplement: Supplementary file 1 [file insects-14-00457-s001.zip › insects-2317506-supplementary.pdf]

Table S1. Insecticide discriminating concentrations for WHO susceptibility bioassays with *Anopheles* mosquitoes [13,14].

Insecticide class/synergist	Test method	Insecticide	Species for which DCs are validated	Discriminating concentration[a]	Exposure period (hour)	Holding period (hour)	Carrier oil/solvent/ surfactant
Pyrethroids	WHO tube test	Alpha-cypermethrin	*An. funestus* s.s., *An. gambiae* s.s., *An. minimus*	0.05% [b]	1 h	24 h	Silicone oil
			An. albimanus, *An. stephensi*	0.30% [b]	1 h	24 h	Silicone oil
		Cyfluthrin	*An. aconitus*, *An. albimanus*, *An. arabiensis*, *An. dirus*, *An. freeborni*, *An. gambiae* s.s., *An. maculatus*, *An. minimus*, *An. stephensi*	0.15%	1 h	24 h	Silicone oil
		Deltamethrin	*An. aconitus*, *An. albimanus*, *An. arabiensis*, *An. dirus*, *An. freeborni*, *An. gambiae* s.s., *An. maculatus*, *An. minimus*, *An. stephensi*	0.05%	1 h	24 h	Silicone oil

		Etofenprox	*An. aconitus*, *An. albimanus*, *An. arabiensis*, *An. dirus*, *An. freeborni*, *An. gambiae* s.s., *An. maculatus*, *An. stephensi*	0.50%	1 h	24 h	Silicone oil
		Lambda-cyhalothrin	*An. aconitus*, *An. albimanus*, *An. arabiensis*, *An. dirus, An. freeborni*, *An. gambiae* s.s., *An. maculatus*, *An. minimus*, *An. stephensi*	0.05%	1 h	24 h	Silicone oil
			An. sacharovi	0.10%	1 h	24 h	Silicone oil
		Permethrin (40:60 cis:trans isomer ratio)	*An. aconitus*, *An. albimanus*, *An. arabiensis*, *An. dirus*, *An, freeborni*, *An. gambiae* s.s., *An. maculatus*, *An. minimus*, *An. stephensi*	0.75%	1 h	24 h	Silicone oil
	WHO bottle bioassay	Transfluthrin	*An. albimanus*, *An. stephensi*, *An. funestus*, *An. minimus*, *An. gambiae*	2 µg/bottle	1 h	24 h	Acetone only

Carbamates	WHO tube test	Bendiocarb	NA	0.10%	1 h	24 h	Olive oil
		Carbosulfan	NA	0.40% [c,d]	1 h	24 h	Olive oil
		Propoxur	NA	0.10% [d]	1 h	24 h	Olive oil
Organochlorines		DDT	NA	4.00%	1 h	24 h	Risella oil
		Dieldrin	NA	4.00%/0.4% [d,e]	1 h	24 h	Risella oil
Organophosphates		Fenitrothion	NA	1.00% [e]	2 h	24 h	Olive oil
		Malathion	NA	5.00%	1 h	24 h	Olive oil
		Pirimiphos-methyl	*An. albimanus*, *An. stephensi*, *An. minimus*, *An. funestus* s.s.	100 mg/m^2 [b]	1 h	24 h	Acetone only
			An. gambiae s.s	170 mg/m^2 [b]	1 h	24 h	Acetone only
Synergist		Piperonyl butoxide	NA	4%	1 h	24 h	Silicone oil
Neonicotinoids	WHO bottle bioassay	Clothianidin	*An. albinamus*, *An. stephensi*	10 µg/bottle	1 h	24 h	Acetone + MERO 800 ppm
			An. funestus s.s., *An. gambiae* s.s.	4 µg/bottle	1 h	24 h	Acetone + MERO 800 ppm
Butenolides		Flupyradifurone	*An. albimanus*	500 µg/bottle	1 h	24 h	Acetone + MERO 200 ppm
			An. stephensi, *An. gambiae* s.s.	60 µg/bottle	1 h	24 h	Acetone + MERO 200

							ppm
			An. funestus s.s., *An. minimus*	100 µg/bottle	1 h	24 h	Acetone + MERO 200 ppm
Pyrroles		Chlorfenapyr	*An. gambiae* s.s., *An. stephensi*, *An. funestus* s.s., *An.albimanus*	100 µg/bottle	1 h	72 h	Acetone only
JH hormone mimics		Pyriproxyfen	*An. gambiae* s.s., *An. stephensi*, *An. funestus* s.s.	100 µg/bottle	1 h	72 h for mortality, 7 d for oviposition inhibition	Acetone only

[a] Tube test: DC as percentage; for pirimiphos-methyl, in mg/m2. Bottle bioassay: DC in µg/bottle (250 mL).

[b] These discriminating concentrations superspeed the tentative concentration presented in the Test procedures for resistance monitoring in malaria vector mosquitoes (second edition) (WHO, [41]).

[c] Tentative, needs confirmation. Based on data published by N'Guessan et al. [39] and Ahoua Alou et al. [38].

[d] Impregnated papers no longer supplied by USM.

[e] Exposure to dieldrin at 0.4% kills susceptible (SS) mosquitoes but not resistant heterozygotes (RS); exposure to dieldrin at 4% kills heterozygotes (RS) but not homozygous (RR) resistant individuals (Rowland et al. [40]).

Table S2. Insecticide discriminating concentrations for WHO susceptibility bioassays with *Aedes* mosquitoes [13,14].

Insecticide class	Test method	Insecticide	Species for which DCs are validated	Discriminating concentrations[a]	Exposure period (h)	Holding period (h)	Carrier oil/solvent/surfactant
Pyrethroids	WHO tube test	Alpha-cypermethrin	*Ae. aegypti*	0.05% [b]	1 h	24 h	Silicone oil
			Ae. albopictus	0.08% [b]	1 h	24 h	Silicone oil
		Deltamethrin	*Ae. aegypti*, *Ae. albopictus*	0.03% [b]	1 h	24 h	Silicone oil
		Lambda-cyhalothrin	*Ae. aegypti*	0.05% [b]	1 h	24 h	Silicone oil
			Ae. albopictus	0.08% [b]	1 h	24 h	Silicone oil
		Permethrin (40:60 cis:trans isomer ratio)	*Ae. aegypti*, *Ae. albopictus*	0.4% [b]	1 h	24 h	Silicone oil
	WHO bottle bioassay	Transfluthrin	*Ae. aegpyti*, *Ae. albopictus*	3 µg/bottle	1 h	24 h	Acetone only
		Metofluthrin	*Ae. aegpyti*, *Ae. albopictus*	1 µg/bottle	1 h	24 h	Acetone only
		Prallethrin	*Ae. aegpyti*, *Ae. albopictus*	30 µg/bottle	1 h	24 h	Acetone only
Carbamates	WHO tube test	Bendiocarb	*Ae. aegypti*, *Ae. albopictus*	0.20%	1 h	24 h	Olive oil
		Propoxur	*Ae. aegypti*	0.10% [c]	1 h	24 h	Olive oil
Organophosphates		Chlorpyrifos-ethyl	*Ae. aegypti*, *Ae. albopictus*	1.00%	1 h	24 h	Olive oil

		Pirimiphos-methyl	*Ae. Aegypti, Ae. albopictus*	60 mg/m^2 [b]	1 h	24 h	Acetone only
		Malathion	*Ae. aegypti*	1.5% [b]	1 h	24 h	Olive Oil
			Ae. albopictus	5% [b]	1 h	24 h	Olive Oil
Neonicotinoids	WHO bottle bioassay	Clothianidin	*Ae. aegypti*	20 µg/bottle	1 h	24 h	Acetone + MERO 1500 ppm
			Ae. albopictus	10 µg/bottle	1 h	24 h	Acetone + MERO 1500 ppm
Butenolides		Flupyradifurone	*Ae. aegpyti, Ae. albopictus*	80 µg/bottle	1 h	24 h	Acetone + MERO 1500 ppm

MERO: 81% rapeseed oil methyl ester (manufactured by Bayer CropScience).

[a] Tube test: DC as percentage. Bottle bioassay: DC in µg/bottle (250 mL).

[b] These discriminating concentrations supersede the tentative concentration presented in the Test procedures for resistance monitoring in malaria vector mosquitoes (second edition) (WHO [41])

[c] Impregnated papers no longer supplied by USM.

[38] Ahoua Alou, L.P.; Koffi, A.A.; Adja, M.A.; Tia, E.; Kouassi, P.K.; Koné, M.; Chandre, F. Distribution of ace-1R and resistance to carbamates and organophosphates in *Anopheles gambiae* s.s. populations from Côte d'Ivoire. *Malar. J.* **2010**, *9*,167.

[39] N'Guessan, R.; Darriet, F.; Guillet, P.; Carnevale, P.; Traore-Lamizana, M.; Corbel, V.; Koffi, A.A.; Chandre, F. Resistance to carbosulfan in *Anopheles gambiae* from Ivory Coast, based on reduced sensitivity of acetylcholinesterase. *Med. Vet. Entomol.* **2003**, *17*, 19-25.

[40] Rowland, M. Behaviour and fitness of HCH/dieldrin resistant and susceptible females of *Anopheles gambiae* and *Anopheles stephensi* in the absence of insecticide. *Med. Vet. Entomol*. **1991**, *5*, 193–206.

[41] World Health Organization. *Test Procedures for Insecticide Resistance Monitoring in Malaria Vector Mosquitoes*, 2nd ed.; World Health Organization：Geneva, Switzerland, 2016. Available online: https://apps.who.int/iris/handle/10665/250677 (accessed on 8 December 2022).

Table S3. Recommended number of mosquitoes to use in the resistance surveillance tests.

Category	Age	Number of concentrations	Number of insects / Concentration
Larval test	L3-L4	5 (+ control)	100 (+ 100 control)
Tube test	3-5 D	1 (+ control)	100 (+ 50 control)
Bottle test	3-5 D	1 (+ control)	100 (+ 50 control)
